# Effect of discontinuous glass fibers on mechanical properties of glass ionomer cement

**DOI:** 10.1080/23337931.2018.1491798

**Published:** 2018-07-31

**Authors:** Sufyan K. Garoushi, Jingwei He, Pekka K. Vallittu, Lippo V. J. Lassila

**Affiliations:** a Department of Biomaterials Science and Turku Clinical Biomaterials Center–TCBC, Institute of Dentistry, University of Turku, Turku, Finland;; b College of Materials Science and Engineering, South China University of Technology, Guangzhou, China;; c City of Turku Welfare Division, Oral Health Care, Turku, Finland

**Keywords:** Mechanical properties, discontinuous fiber reinforcement, glass ionomer cement

## Abstract

Aim: This study investigated the reinforcing effect of discontinuous glass microfibers with various loading fractions on selected mechanical properties of self-cure glass ionomer cement (GIC).

Method: Experimental fiber reinforced GIC (Exp-GIC) was prepared by adding discontinuous glass microfiber (silane/non-silane treated) of 200–300 µm in length to the powder of self-cure GIC (GC Fuji IX) with various mass ratios (15, 20, 25, 35, and 45 mass%) using a high speed mixing device. Flexural strength, flexural modulus, work of fracture, compressive strength and diametral tensile strength were determined for each experimental and control materials. The specimens (*n* = 8) were wet stored (37 °C for one day) before testing. Scanning electron microscopy equipped with energy dispersive spectrometer was used to analysis the surface of silanized or non-silanized fibers after treated with cement liquid. The results were analyzed with using multivariate analysis of variance MANOVA.

Results: Fiber-reinforced GIC (25 mass%) had significantly higher mechanical performance of flexural modulus (3.8 GPa), flexural strength (48 MPa), and diametral tensile strength (18 MPa) (*p* < .05) compared to unreinforced material (0.9 GPa, 26 MPa and 8 MPa). No statistical significant difference in tested mechanical properties was recorded between silanized and non-silanized Exp-GIC groups. Compressive strength did not show any significant differences (*p* > .05) between the fiber-reinforced and unreinforced GIC.

Conclusion: The use of discontinuous glass microfibers with self-cure GIC matrix considerably increased the all of the studied properties except compressive strength.

## Introduction

An increasing demand for direct filling materials in dentistry has been supported by changes in restorative techniques. The trend of adhesively bonded restorations saves sound tooth structure and is compatible with prophylactic concepts. Preserving and stabilizing hard tooth tissues by direct filling techniques are favored over macromechanically styled, destructive preparations with amalgam or indirect restorative materials [1,2].

Nowadays, glass ionomer cements (GICs) are used in many dental applications due to several unique advantages among restorative materials [3]. Their benefits include fluoride release and uptake, biocompatibility, favorable thermal expansion, and chemical bonding to tooth structure [4]. In addition, GICs have shown their potential in other medical areas, such as orthopedic surgery as bone cement [5]. On the other hand, poor mechanical properties, such as low flexural strength, fracture resistance and wear, limit their wider use in dentistry as a permanent filling material in stress-bearing areas [2,3]. In the posterior dental region, GICs are mostly used as a temporary filling material or base [2]. Reinforcement of glass ionomer restorative materials is essential and many researchers have focused on improving the mechanical properties by adding various filler types to the GIC powder component. The fillers used included metallic powders, hydroxyapatite powders, bioactive glass particles, nanoclay and discontinuous glass fibers [5–16].

In dentistry, fiber reinforcement has become clinically recognized technology that could offer a new affordable option for both patients and clinicians. Discontinuous microfiber reinforcement, i.e. reinforcing fibers with diameter from a few micrometers to twenty micrometers with high aspect ratio, are utilized in many fields of technical application as well as in dentistry and medicine [17]. However they have not been studied to a larger extent with GICs [3]. Although, little information about the use of discontinuous glass fibers with GIC composites are available, hence further investigation is required in order to produce a material with improved properties [3]. Many of the properties of fiber composites are strongly dependent on microstructural variables such as fiber diameter, fiber length, fiber orientation, fiber adhesion and fiber loading [17]. Therefore, the intent of this study was to evaluate the effect of microfibers with various loading fractions and surface treatments on selected mechanical properties of self-cure GIC composite.

## Materials and methods

### Production of experimental discontinuous fiber reinforced GICs

The discontinuous E-glass fibers having length scale of 200–300 micrometer (Ø6 μm), so-called discontinuous E-glass microfibers, as-received either silanized (3-[Trimethoxysilyl] propyl methacrylate, MPS) or non-silanized were used in this study. Experimental fiber reinforced GIC composites were prepared by adding discontinuous glass microfiber to the glass powder (fluoroaluminosilicate glass) of commercial self-cure GIC (GC Fuji IX, shade A3, Tokyo, Japan) with various mass ratios (15, 20, 25, 35, and 45 mass%). The mixing was performed by using a high speed mixing machine until a homogeneous powder mixture was obtained (Hauschild Speed Mixer DAC 400.1, 3500 rpm). Homogeneous mixture was checked manually by using a hand-plastic instrument and ascertained by light microscope (Leica, Wild Herbrugg, Switzerland) at a magnification of 6.5×. Finally, the materials produced were reinforced powders with various mass ratios of either silanized or non-silanized discontinuous glass microfibers which were compared with unmodified material. The reinforced glass powder and the cement liquid (modified polyacrylic acid & water) were mixed and manipulated according to the manufacturers’ instructions.

### Mechanical tests

Flexural strength (FS) and modulus (FM) were determined by conducting a 3-point bending specimens (2 × 2 × 25 mm^3^) from each tested material. The recommended powder/liquid ratio was dispensed on a glass plate. Then this powder was mixed into the liquid using a metal spatula. The mixing time did not exceed 1 min and the working time was in the range of 2–3 min. Bar-shaped specimens were made in a half-split stainless steel mold between transparent Mylar sheets and a glass slide. Specimens were kept in their molds for 30 min before they were carefully removed.

The specimens from each group (*n* = 8) were stored wet (wrapped in wet tissue) at 37 °C for 24 h before testing. The three-point bending test was conducted (test span: 20 mm, cross-head speed: 1 mm/min, loading pin: 2 mm diameter). All specimens were loaded in a material testing machine (model LRX, Lloyd Instruments Ltd., Fareham, England) and the load-deflection curves were recorded with computer software (Nexygen 4.0, Lloyd Instruments Ltd., Fareham, England).

Flexural strength (ơ_f_) and flexural modulus (E_f_) were calculated from the following formula (ISO 1992):

$${\text{ơ}}_{\text{f}}={\text{ 3F}}_{\text{m}}\text{L}/\left({\text{2bh}}^{\text{2}}\right)$$

$${\text{E}}_{\text{f}}={\text{ SL}}^{\text{3}}/\left({\text{4bh}}^{\text{3}}\right)$$

where F_m_ is the applied load (N) at the highest point of a load-deflection curve, L is the span length (20 mm), b is the width of test specimens and h is the thickness of test specimens. S is the stiffness (N/m). S = F/d and d is the deflection corresponding to load F at a point in the straight-line portion of the trace.

Work of fracture (the energy required to fracture the 3-point bending specimen) was calculated from the area under the load-displacement curve of specimens and reported in units of J/m^2^.

Diametral tensile strength (DTS) and compressive strength (CS) were determined on cylindrical specimens (4 mm in diameter and 6 mm in height) that were prepared in the same way according to ISO 4104 [18]. The manipulation of the GIC materials was the same as mentioned earlier. For the DTS, each specimen was placed with its longitudinal side between the platens of the testing machine. The specimens were loaded in compression until failure at a crosshead speed of 1 mm/min. The length and diameter of each specimen were measured before testing with a digital caliper, and the DTS was calculated based on specimen length, diameter, and peak load. For the CS, the same testing performances were used, with the specimen being placed with the flat end on the supporting plate. The flat ends were fixed between the platens of the testing machine. In this case, a compressive load was applied axially until failure. The CS was determined using the peak load at fracture and the diameter of the specimen.

DTS in megapascals (MPa) was calculated using the Equation (1): T = 2 F/π L D [18], where: T is the diametral tensile strength, F is the maximum applied load in newtons (N); D is the diameter of the specimens in mm and L is the length of the specimen in mm. CS was calculated in megapascals (MPa) using the Equation (2): *P* = 4 F π D2 [18], where: P is the compressive strength, F is the maximum applied load in newtons (N) and D is the diameter of the specimen in mm.

### Microscopic analysis of the fiber-matrix interface

The silanized or non-silanized discontinuous glass microfibers were mixed with cement liquid and stored for a period of time until the mixtures became rigid. Then, the mixtures were repeatedly reflux extracted by Soxhlet extractor for 3 days, ethanol was used as extraction medium. The remain substances were dried at 60 °C under vacuum until the mass keeping stable. Scanning electron microscopy (EVO18, Carl Zeiss AG, Göttingen, Germany) equipped with energy dispersive spectrometer (EDS) was used to observe the morphology of dried substances and investigate mass ratio between C and Si on the surface of substances.

### Fiber length measurement

The analysis of discontinuous fiber length distribution was done for the fiber reinforced GICs. Scope of powder taken on glassware, then 2 ml of tetrahydrofuran (THF, Riedel de Haen, Puriss 99.9%, Lot; 404750) was added on to the glassware using a Pasteur-pipette. When it was observed that the fibers had started to separate, the THF was removed with a Pasteur pipette. A total of 3 times this procedure was repeated and afterwards when the THF had vaporized and fibers were dried, they were photographed with a stereo-microscope (Heerbrugg M3Z, Switzerland) at a magnification of 6.5×. The resulting photos were then processed with the Image-J processing program to determine the final lengths of the fibers. The total number of fibers taken into the calculation was 500.

### Statistical analysis

The data of FS, DTS and CS were statistically analyzed with SPSS version 23 (SPSS, IBM Corp.) using multivariate analysis of variance (MANOVA) at the *p* < .05 critical value, followed by a Tukey HSD *post hoc* test to determine the differences between the groups.

## Results

**Figure 1.**
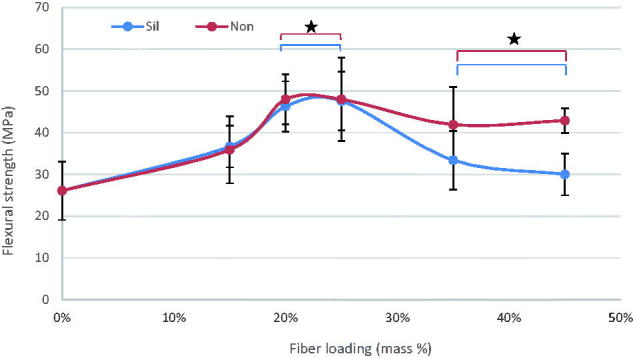
Influence of increasing fraction of discontinuous glass microfiber on flexural strength of investigated self-cure GIC material. Groups joined by a line are not significantly difference (*p > .05) (Sil = silanized fibers, Non = not silanized fibers).

**Figure 2.**
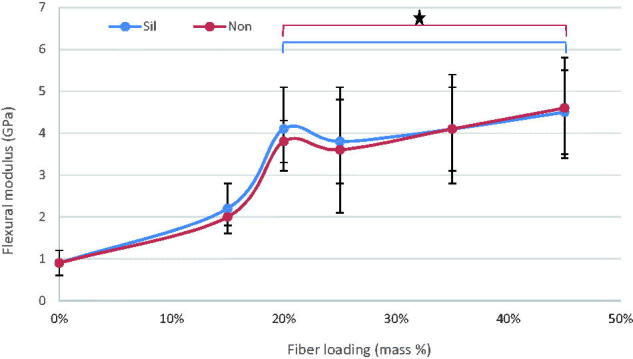
Influence of increasing fraction of discontinuous glass microfiber on flexural modulus of investigated self-cure GIC material. Groups joined by a line are not significantly difference (*p > .05) (Sil = silanized fibers, Non = not silanized fibers).

**Figure 3.**
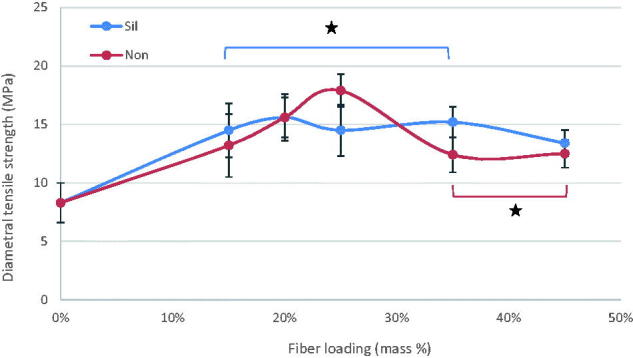
Influence of increasing fraction of discontinuous glass microfiber on diametral tensile strength of investigated self-cure GIC material. Groups joined by a line are not significantly difference (*p > .05) (Sil = silanized fibers, Non = not silanized fibers).

**Figure 4.**
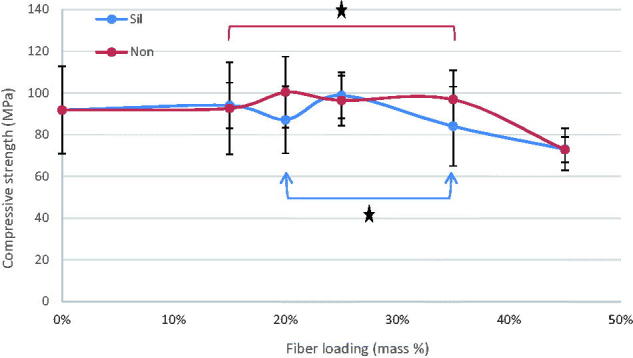
Influence of increasing fraction of discontinuous glass microfiber on compressive strength of investigated self-cure GIC material. Groups joined by a line are not significantly difference (*p > .05) (Sil = silanized fibers, Non = not silanized fibers).

The mean values of flexural strength (FS), flexural modulus (FM), diametral tensile strength (DTS), and compressive strength (CS) for tested materials with standard deviations (SD) are summarized at Figures 1–4. In general, the discontinuous microfiber reinforcement (silane/non-silane) significantly improved most of the tested mechanical properties of self-cure GIC composites (*p* < .05) with no difference between silanized and non-silanized groups. The data showed that by increasing the fiber mass ratios, regardless of fiber surface treatment type, the mechanical properties increased (*p* < .05). Compressive strength was the only property which did not show any significant differences (*p* > .05) between the fiber reinforced and unmodified commercial self-cure GICs. Tukey HSD *post hoc* revealed that Exp-GIC 20 mass% and Exp-GIC 25 mass% had statistically significantly higher FS (48 MPa) and DTS (18 MPa) than all other tested GIC materials (*p* < .05). On the other hand, Exp-GIC 45 mass% displayed the highest only with FM (4.6 GPa) among all tested groups.

**Figure 5.**
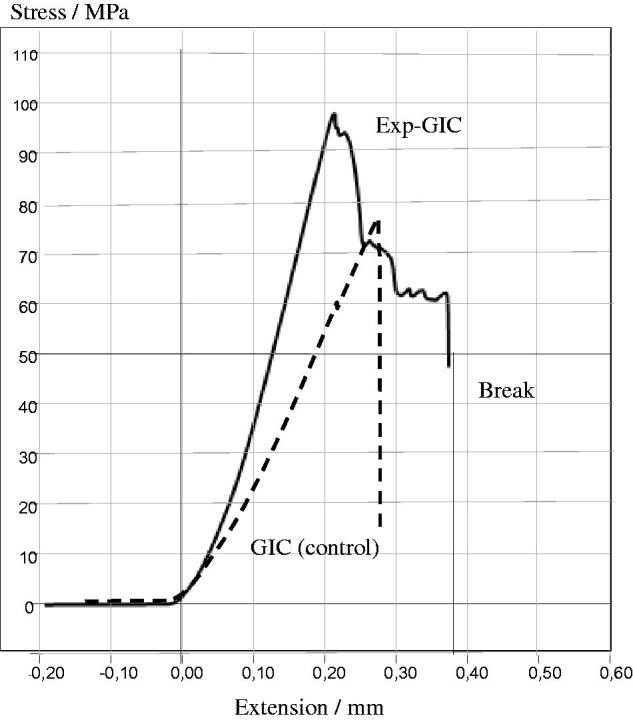
Graph illustrating typical load-strain curves obtained from compression test of investigated self-cure GIC (control, dashes line) and fiber reinforced GIC (Exp-GIC, straight line).

**Figure 6.**
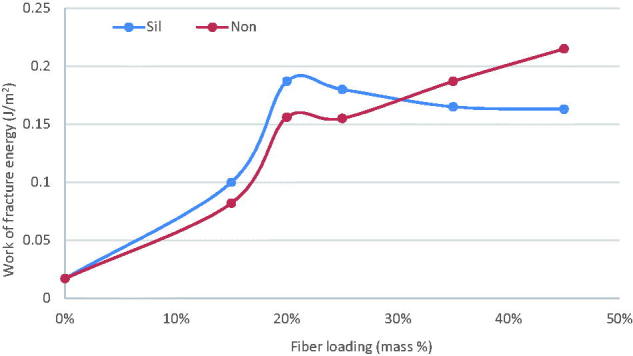
Influence of increasing fraction of discontinuous glass microfiber on work of fracture energy of investigated self-cure GIC material during 3-point bending test (Sil = silanized fibers, Non = not silanized fibers).

The total energy release (work of fracture) of experimental and unmodified commercial GIC composites was calculated from the area under the load-displacement curves, shown in Figure 5. The contribution of microfiber plays a major role in increasing the work of fracture energy of Exp-GIC in comparison to unmodified commercial GIC. Figure 6 illustrates a pronounced increase in the energy of fracture as the percentage of discontinuous glass microfiber was increased.

**Figure 7.**
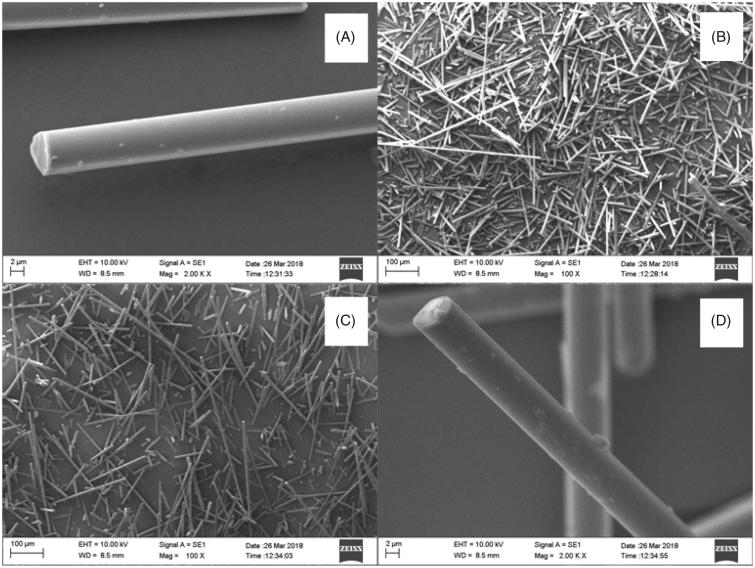
SEM micrographs of non-silanized (A: ×100 magnification, scale bar = 100 µm; B: ×2000 magnification, scale bar = 2 µm) and silanized fibers (C: ×100 magnification, scale bar = 100 µm; D: ×2000 magnification, scale bar = 2 µm) after being mixed with cement liquid followed extraction.

**Figure 8.**
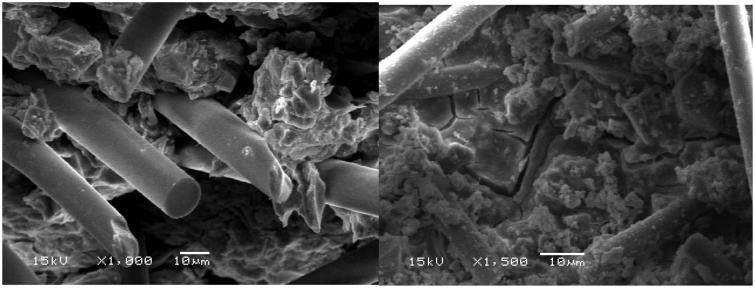
SEM micrographs of fracture surface demonstrate random orientation and pull-out of discontinuous glass microfiber reinforced self-cure GICs.

**Figure 9.**
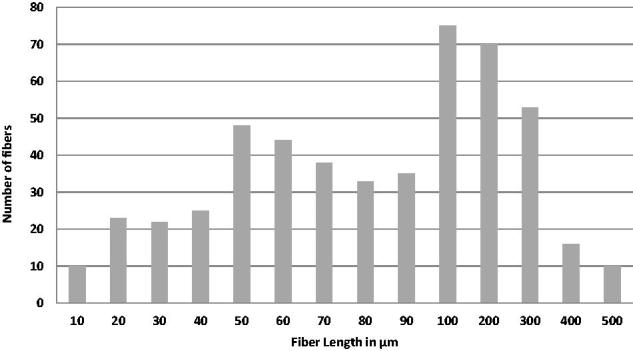
Length distribution of discontinuous microfibers in the reinforced GIC powder.

The SEM micrographs (Figure 7) of extracted mixtures of glass microfibers and cement liquid showed that all of remain substances were fibers, and the morphologies of silanized and non-silanized fibers had no differences. The EDS results (Table 1) showed that, after being mixed with cement liquid followed by extraction, the mass ratio between C and Si on the surface of non-silanized fibers was higher than that on the surface of silanized fibers. SEM analysis of the tested 3-point bending specimens showed random orientation and protruded (pullout) fiber ends at fracture surfaces of discontinuous glass microfiber reinforced self-cure GIC composites (Figure 8). Figure 9 showed the fiber length distribution of the discontinuous microfiber reinforced GICs.

**Table 1.** Mass ratio between C and Si on the surfaces of non-silanized and silanized fibers after being mixed with cement liquid followed by extraction.

Fibers	Mass ratio between C and Si
Non-silanized	7.3
Silanized	0.9

## Discussion

The key target of this investigation was to improve the mechanical properties of self-cure GIC that is already used in dental clinics as a restorative dental material. The reinforcing was made by adding discontinuous glass microfiber (Ø6 μm) as fillers in 200–300 µm length, to GIC powder (with various mass%). Maximum fiber fraction was defined based on handling and mixing characteristics. With more than 45 mass% of discontinuous glass microfiber, the material became more fibrous and was not possible to mix.

Our results demonstrated improved mechanical properties of the discontinuous glass microfiber reinforced self-cure GIC composite. As the mass fraction of fibers increased, the mechanical performance of the GIC increased, which is in good agreement with the literature findings [1,7,8,19,20]. However, the increase of tested mechanical properties was achieved for 20 & 25 mass% microfiber loading as shown in Figures 1–4. Fiber loading is reported to be limited within a small fiber fraction. It is limited by a critical fiber volume that has to be exceeded to result in an increased strength or toughness [21] and by a maximum fiber loading above which large microstructural defects or voids might formed [22].

The major goal to improve mechanical properties has been reached successfully, as the absorbed energy during the fracture process of microfiber reinforced GIC composite could be dramatically improved compared with unmodified GICs (Figures 5 and 6).

Sufficient adhesion between fiber and matrix provides good load transfer between the two ingredients, which ensures that the load is transferred to the stronger fiber and this is how the fiber actually works as reinforcement. However, if the adhesion is not strong and if any voids appear between the fiber and the matrix, these voids may act as initial fracture sites in the matrix and facilitate the breakdown of the material. That is why adhesion between the fiber and the GIC matrix is significant for the mechanical performance and the longevity of restorations [17]. To the authors' knowledge, comparative data between silane and non-silane glass fiber reinforcing system with GIC is not documented in dental literature. In this study, fibers were received either silanised or non-silanised from the manufacturer, the used silane is compatible with both acrylate and epoxy resins. When fiber surface silane is esterified with polyacrylic acid, a polymer with functional double bonds is established [23,24]. Lassas et al. showed the formation of ion-bond polyacrylates on the silanised glass fiber surface and the amount of ions exchanged increases with increased acid concentration [24]. On the other hand, silanol groups can react with the OH- groups and they form a siloxane bond and with metal ions can form a metal-siloxane bond but they are weak and have poor hydrolytic stability [23]. Previous studies stated that the presence of aluminum and calcium ions on glass fiber surface makes it possible to obtain a reactive layer at the interface between the glass fiber surface and polyacrylic acid [1,24]. Lohbauer et al. showed a distinct reactive layer (2–20 µm in thickness) at the interface between the GIC matrix and glass fiber formed during the setting process [1]. However, the ion leaching and reactivity of the glass fiber surface might be affected by the fiber processing and silanization [1]. Wilson stated in his work that the interface between the silica gel layer around the glass core tends to be weak and also that the failure’s origin was located at the interface [25]. From this point of view, the great increase in both fracture load and energy of discontinuous glass microfiber reinforced GIC composites, could be explained by the chemical adhesion between the fibers and GIC matrix. This seems to have enhanced the ability of the material to resist the fracture crack propagation by increasing the behavior of individual fiber as a crack stopper. Even though, we were not able to distinguish a reactive layer between the GIC matrix and glass fiber surface (silane/non-silane) by using scanning electron microscopy. In this work, we attempted to study the adhesion between silane/non-silane glass fibers and GIC matrix by SEM equipped with EDS, and the fibers were treated with cement liquid and extracted by ethanol. Theoretically, if the adhesion between fibers and polyacrylic acid is strong, there will be more C content on the surface of fibers, since polyacrylic acid has higher C content than fibers. The results showed that the ions exchange or reaction between non-silane fibers and polyacrylic acid was greater than that of silane fibers (Table 1). However, there was no significant differences on mechanical properties between silane fibers reinforced and non-silane fibers reinforced GIC composite, this might be explained that the adhesion between polyacrylic acid and fibers was still too weak.

Since the load bearing capacity of the brittle materials such as GIC is grater in compression than in tension. GIC fails by crack propagation that is favored by tensile rather than compressive loading. The mechanism leading to failure under compressive loading remains more complex than that observed in tensile strength test, since the cracks propagate parallel to the compression axis as the lateral deformation increases during test progression [26]. While, flexural strength test the material under both compressive and tensile loading [27]. Dowling et al. demonstrated the validity of the three-point and biaxial flexural tests for measuring GIC strength in comparison with compressive fracture test which they claimed it was not valid for predicting the performance of GIC [26]. According to them, compressive fracture test has been demonstrated through the literature to be invalid measure of ’strength’ and offers no advantages in the context of the strength data it generates when compared with three-point or biaxial flexural testing.

According to Darvell BW, because of shear stresses at contact area between applied load and specimens and being sensitive to inter-operator variability, the validity of the diametral tensile test for brittle materials was also comprehensively undermined [28]. Even though the validity of some testing methodologies like compressive fracture and diametral tensile tests is challenged in the literature, still many research groups using these tests regularly for GIC and still compressive fracture test is the only strength test specified for GIC in the international organization for standardization (ISO 9917–1:2003) [29].

In general, the effect of discontinuous fiber reinforcement on the flexural properties and diametral tensile tests were observable and more obvious than in the compression test, where fibers were oriented most likely in the same load direction [30]. It has been shown early on that fiber orientation is an important factor influencing the mechanical properties of fiber reinforced composite [31].

In this study, the mean mechanical properties values (FS, FM, CS and DTS) values of reinforced self-cure GIC composite, were different from those of fiber reinforced GIC composites reported in the literature [1,6,19,32]. On the other hand, this study confirms that the mean mechanical values reported for the commercial GICs without reinforcement are within the range of data previously reported [7,9,33,34]. With respect to fiber incorporation in GIC powders, the aspect ratio (fiber length:diameter), chemical composition, reinforcing fiber fraction, composition of the GIC powder and liquid and the powder:liquid mixing ratios employed were markedly for the studies reporting fiber reinforcement of GICs, such that a direct comparison under controlled conditions was not possible [3].

In order for a fiber to act as an effective reinforcement for GIC matrix, stress transfer from the matrix to the fibers is essential. This is achieved by having a fiber length equal to or greater than the critical fiber length [17]. When the discontinuous glass fibers were mixed with GIC powder at high speed, the rupture of fibers happened and fiber lengths were different before and after the mixing procedure. To have a better understanding, the analysis of fiber length distribution was carried out to investigate the real final lengths of discontinuous glass microfibers in the GIC powder.

The fiber length distribution was approximately between 50 and 300 µm (Figure 9), which is lower than the reported range of the critical fiber length and desired aspect ratio [6,7]. By taking into account the aspect ratio of discontinuous glass fibers, the critical fiber length has been suggested to be as much as 50 times the diameter of the fiber [14]. The diameter of glass fiber used in the present study was in the range of 6 μm and the critical length should be, therefore around 300 µm. Since fiber damage occurs during mixing, the experimental fiber length did not reach the critical fiber length. Thus, a further increase of the reinforcement might be expected from using fibers of a higher aspect ratio.

Recent systemic review by Heintz et al. showed that three-point flexural test and fracture toughness test being mostly correlated with clinical fracture and wear of restorative materials and no correlations were observed between clinical outcomes and flexural modulus or compressive strength of these materials [35]. Another study by Bijelic-Donova et al. showed that toughness of the material appears to be a useful predictor of clinical performance and consequently, the selection of the restorative material should be based on its toughness [36]. Considering the difference in toughness (work of fracture energy) and flexural strength values between the experimental reinforced GIC composites and the restorative unmodified GICs, it appears that fiber reinforced GIC composite might be applied as restorative material in high stress bearing areas.

In this investigation the effect of water on the materials was not studied. It has been previously reported that exposure of GIC to water improved the surface with respect to crack initiation under tensile loading [9,37,38]. A visible increase in strength with time stored in water was seen with GICs regardless of the presence or absence of glass fibers [9,37]. This was explained by the maturation of the cement matrix which became more rigid with time. Also, it has been stated that there is a potential deteriorative effect of water to the interfacial adhesion between the matrix to the E-glass fibers through rehydrolysis of silane coupling agent [15,39]. Another feature that we have not included in this study, is the effect of fiber loading on the setting time of cement specimens. Kobayashi et al. showed that by increasing the fiber mass ratios in GIC powder, the setting time became longer. This change in setting kinetics might be explained by the low reactivity and ion-leaching between glass fiber surface and GIC matrix [33]. However, this work and that of Kobayashi et al. failed to consider the effect of powder to liquid ratio upon replacing high surface area powder by lower reactive fibers. This effect will certainly reduce the number of Al^3+^ ions available to assist with the cross linking of the polyacrylic acid. This may partially account for the decreasing strength of Exp-GIC loaded with more than 25 mass% of glass microfiber.

There are still unclear issues that need to be known regarding discontinuous glass microfiber reinforced GIC composite, the biocompatibility, florid release, wear resistance, the bond strength to tooth structures, void content and mixing technique. Therefore, further research is needed and an assessment of optimizing the formulation of this novel discontinuous glass reinforced GIC composites is in progress.

## Conclusion

Based on the results of the present study, one could conclude that the incorporation of discontinuous glass microfiber with self-cure GIC composite resulted in a desirable mechanical performance of flexural modulus (3.8 GPa), flexural strength (48 MPa), and diametral tensile strength (18 MPa) compared to particulate GICs used (0.9 GPa, 26 MPa and 8 MPa).
